# Supplementary material for: Heat adaptation of phage T7 under an extended genetic code
Source: Virus Evol. 2021 Dec 1;7(2):veab100. doi: 10.1093/ve/veab100 (PMC8923235; doi:10.1093/ve/veab100)
Supplement: veab100_Supp [file veab100_supp.zip › Sequencing_Analysis.zip!/Sequencing_Analysis/A1/evidence/SNP_14.html]

BRESEQ :: Evidence


| Predicted mutation | | | | | | |
| --- | --- | --- | --- | --- | --- | --- |
| evidence | seq id | position | mutation | annotation | gene | description |
| RA | NC\_001604.1 | 36,054 | T→G | intergenic (–/–) | – / – | –/– |

| Read alignment evidence... | | | | | | | | | | | |
| --- | --- | --- | --- | --- | --- | --- | --- | --- | --- | --- | --- |
|  | seq id | position | | ref | new | freq | score (cons/poly) | reads | annotation | genes | product |
| \* | NC\_001604.1 | 36,054 | 0 | T | G | 97.5% | 11736.4 / 3.8 | 3858 | intergenic (–/–) | *–/–* | –/– |
| Reads supporting (aligned to +/- strand):  **ref** base T (45/50);  **new** base G (2235/1521);  **total** (2282/1576) | | | | | | | | | | | |
| Fisher's exact test for biased strand distribution *p*-value = 1.99e-02 | | | | | | | | | | | |
| Kolmogorov-Smirnov test that lower quality scores support variant *p*-value = 1.00e+00 | | | | | | | | | | | |

Only 100 of 3858 total aligned reads displayed.  

`CCGCATCCGACAAGGCGCACTATATTCTATCTAAAGATGGTAACAGGAATAACTGGTACATTGGTAGAGGGTCAGATAACAACAATGACTGTACCTTCCACTCCTATGTACATGGTACGACCTTAACACTCAAGCAGGACTATGCAGTAGTTAACAAACACTTCCACGTAGGTCAGGCCGTTGTGGCCACTGATGGTAATATTCAAGGTACTAAGTGGGGAGGTAAATGGCTGGATGCTTACCTACGTGACAGCTTCGTTGCGAAGTCCAAGGCGTGGACTCAGGTGTGGTCTGGTAGTGCTGGCGGTGGGGTAAGTGTGACTGTTTCACAGGATCTCCGCTTCCGCAATATCTGGATTAAGTGTGCCAACAACTCTTGGAACTTCTTCCGTACTGGCCCCGATGGAATCTACTTCATAGCCTCTGATGGTGGATGGTTACGATTCCAAATACACTCCAACGGTCTCGGATTCAAGAATATTGCAGACAGTCGTTCAGTACCTAATGCAATCATGGTGGAGAACGAGTAATTGGTAAATCACAAGGAAAGACGTGTAGTCCACGGATGGACTCTCAAGGAGGTACAAGGTGCTATCA  >  NC_001604.1/35756‑36352`  

                                                                                                                                                                                                                                                                                                          `|`                                                                                                                                                                                                                                                                                                            
   `cATCCGACAAGGCGCACTATATTCTATCTAAAGATGGTAACAGGAATAACTGGTACATTGGTAGAGGGTCAGATAACAACAATGACTGTACCTTCCACTCCTATGTACATGGTACGACCTTAACACTCAAGCAGGACTATGCAGTAGTTAACAAACACTTCCACGTAGGTCAGTCCGTTGTGGCCACTGATGGTAATATTCAAGGTACTAAGTGGGGAGGTAAATGGCTGGATGCTTACCTACGTGACAGCTTCGTTGCGAAGTCCAAGGCGTGGACTCAGGTGTGGTCTGGTAGGGCTgg                                                                                                                                                                                                                                                                                                       >  1:342433/1‑301 (MQ=255)`  
                 `gaCTATAGTCTATCTAAAGATGGTAACAGGAATAACTGGTACATTGGTAGAGGGTCAGATAACAACAATGACTGTACCTTCCACTCCTATGTACATTGTACGACCTTAACACTCAAGCAGGACTATGCAGTAGTTAACAAACACTTCCACGTAGGTCAGGCCGTTGTGGCCACTGATGGTAATATTCAAGGTACTAAGTGGGGAGGTAAATGGCTGGATGCTTACCTACGTGACAGCTTCGTTGCGAAGTCCAAGGCGTGGACTCAGGTGTGGTCTGGTAGGGCTGGCGGTGGGGTAAgtg                                                                                                                                                                                                                                                                                         <  1:489375/300‑1 (MQ=255)`  
                 `cACTATAGTCTATACAAAGATGGTAACAGGAATAACTGGTACATTGGTAGAGGGTCAGATAACAACAATGACTGTACCTTCCACTCCTATGTACATGGTACGACCTTAACACTCAAGCAGGACTATGCATTAGTTAACAAACACTTCCACGTAGGTCAGGCCGTTGTGGCCACTGATGGTAATATTCAAGGTACTAAGTGGGGAGGTAAATGGCTGGATGCTTACCTACGTGACAGCTTCGTTGCGAAGTCCAAGGCGTGGACTCAGGTGTGGTCTGGTAGGGCTGGCGGTGGGGTAAgtg                                                                                                                                                                                                                                                                                         <  1:171479/301‑1 (MQ=255)`  
                  `aCTATATTCTATCTAAAGATGGTAACAGGAATAACTGGTACATTGGTAGAGGGTCAGATAACAACAATGACTGTACCTTCCACTCCTATGTACATGGTACGACCTTAACACTCAAGCAGGACTATGCAGTAGTTAACAAACACTTCCACGTAGGTCAGGCCGTTGTGGCCACTGATGGTAATATTCAAGGTACTAAGTGGGGAGGTAAATGGCTGGATGCTTACCTACGTGACAGCTTCGTTGCGAAGTCCAAGGCGTGGACTCAGGTGTGGTCTGGTAGGGCTGGCGGTGGGGTAAgtgc                                                                                                                                                                                                                                                                                        >  1:390348/1‑300 (MQ=255)`  
                   `cTATATTCTATCTAAAGATGGTAACAGGAATAACTGGTACATTGGTAGAGGGTCAGATAACAACAATGACTGTACCTTCCACTCCTATGTACATGGTACGACCTTAACACTCAAGCAGGACTATGTAGTAGTTAACAAACACTTCCACGTAGGTCAGTCCTTTGTGGCCACCGATGGTACTATTCAAGGTACTAAGCGGGGAGGTAAATGGCTGGATGCTTACCTACGTGACAGCTTCGTTGCGAAGTCAAAGGCGTGGACTCAGGTGTGGTCTGGGAGTGccccgtatacacatctccgg                                                                                                                                                                                                                                                                                       >  1:502801/1‑282 (MQ=255)`  
                   `cTATATTCTATCTAAAGATGGTAACAGGAATAACTGGTACATTGGTAGAGGGTCAGATAACAACAATGACTGTACCTTCCACTCCTATGTACATGGTACGACCTTAACACTCAAGCAGGACTATGCAGTAGTTAACAGACACTTCCACGTAGGTCAGGCCGTTGTGGCCACTGATGGTAATATTCAAGGTACTAAGTGGGGAGGTAAATGTCTGGATGCTTACCTACGTGACAGCTTCGTTGCGAAGTCCAAGGCGTGGTCTCAGGTGTGGTCTGGTAGGGCTGGCGGTGGGGTAAGtgtg                                                                                                                                                                                                                                                                                       >  1:244534/1‑301 (MQ=255)`  
                     `atatTCTATCTAAAGATGGTAACAGGAATAACTGGTACATTGGTAGAGGGTCAGATAACAACAATGACTGTACCTTCCACTCCTATGTACATGGTACGACCTTAACACTCAAGCAGGACTATGCAGTAGTTAACAAACACTTCCACGTAGGTCAGGCCGTTGTGGCCACTGATTGTAATATTCAAGGTACTAAGTGGGGAGGTAAATGGCTGGATGCTTACCTACGTGACAGCTTCGTTGCGAAGTCCAAGGCGTGGACTCAGGTGTGGTCTGGTAGTGCTGGCGGTGGGGTAAGTGTGAc                                                                                                                                                                                                                                                                                     >  1:184580/1‑301 (MQ=255)`  
                       `atTCTATCTAAAGATGGTAACAGGAATAACTGGTACATTGGTAGAGGGTCAGATAACAACAATGACTGTACCTTCCACTCCTATGTACATGGTACGACGTTAACACTCAAGCAGGACTATGCAGTAGTTAACAAACACTTCCACGTAGGTCAGGCCGTTGTGGCCACTGATGGTAATATTCAAGGTACTAAGTGGGGAGGTAAATGGCTGGATGATTACCTACGTGACAGCTTCGTTGCGAAGTACAAGGCGTGGACTCAGGTGTGGACTGGTAGGGCTGGAGGTGGGGGAActgtcccgt                                                                                                                                                                                                                                                                                   >  1:328554/1‑296 (MQ=255)`  
                          `ctatctaAAGATGGTAACAGGAATAACTGGTACATTGGTAGAGGGTCAGATAACAACAATGACTGTACCTTCCACTCCTATGTACATGGTACGACCTTAACACTCAAGCAGGACTATGCAGTAGTTAACAAACACTTCCACGTAGGTCAGGCCGTTGTGGCCACTGATGGTAATATTCAAGGTACTAAGTGGGGAGGTAAATGGCTGGATGCTTACCTACGTGACAGCTTCGTTGCGAAGTCCAAGGCGTGGACTCAGGTGTGGTCTGGTAGGGCTGGCGGTGGGGTAAGTGTGACTGttt                                                                                                                                                                                                                                                                                <  1:411334/301‑1 (MQ=255)`  
                            `atctaAAGATGGTAACAGGAATAACTGGTACATTGGTAGAGGGTCAGATAACAACAATGACTGTACCTTCCACTCCTATGTACATTGTACGACCTTAACACTCAAGCAGGACTATGCAGTAGTTAACAAACACTTCCACGTAGGTCAGGCCGTTGTGGCCACTGATGGTAATATTCAAGGTACTAAGTGGGGAGGTAAATGGCTGTATGCTTACCTATGTGACAGATTCGTTGCGAAGTCCAAGGCGTGGACTCAGGTGTGGTCTGGTAGGGCTGGCGGTGGGGGAAGGGTGGCGTTTTca                                                                                                                                                                                                                                                                              >  1:133691/1‑301 (MQ=255)`  
                               `agatgtgtataagagacagaaCTGGTACATTGGTAGAGGGTCAGATAACAACAATGACTGTACCTTCCACTCCTATGTACATGGTACGACCTTAACACTCAAGCAGGACTATGCAGTAGTTAACAAACACTTCCACGTAGGTCAGGCCGTTGTGGCCACTTATGGTAATATTCAAGGTACTAAGTGGGGAGGTAAATGGCTGGATGCTTACCTACGTGACAGCTTCGTTGCGAAGTCCAAGGCGTGGACTCAGGTGTGGTCTGGTAGGGCTGGCGGTGGGGTAAGTGTGACTGTTTCACAg                                                                                                                                                                                                                                                                           <  1:179645/282‑1 (MQ=255)`  
                                       `gTAACAGGAATAACTGGTACATTGGTAGAGGGTCAGATAACAACAATGACTGTACCTTCCACTCCTATGTACATGGTACGACCTTAACACTCAAGCAGGACTATGCAGTAGTTAACAAACACTTCTACGTAGGTCAGGCCGTTGTGGCCACTGATGGTAATATTCAAGGTACTAAGTGGGGAGGTAAATGGCTGGATGCTTACCTACGTGACAGCTTCGTTGCGAAGTCCAAGGCGTGGACTCAGGTGTGGTCTGGTAGGGCTGGCGGTGGGGTAAGTGTGACTGTTTCACAGGATCTCCg                                                                                                                                                                                                                                                                   <  1:346056/301‑1 (MQ=255)`  
                                                   `ataagagacagggTAGAGGGTCAGATAACAACAATGACTGTACCTTCCACTCCTATGTACATGGTACGACCTTAACACTCAAGAAGGACTATGCAGTAGTTAACAAACACTTCCACGTAGGTCAGGCCGTTGTGGCCACTGATGGTAATATTCAAGGTACTAAGTGGGGAGGTAAATGGCTGGATGCTTACCTACGTGACAGCTTCGTTGCGAAGTCCAAGGCGTGGACTCAGGTGTGGTCTGGTAGGGCTGGCGGTGGGGTAAGTGTGACTGTTTCACAGGATCTCCGCTTCCGAAatat                                                                                                                                                                                                                                                       <  1:356862/290‑1 (MQ=255)`  
                                                       `agatgtggataagagaaaggATAACAACAAGGACTGTACCTTCCACTCCTATGTACATGGTACGACCTTAACACTCAAGCAGGACTATGCAGTAGTTAACAAACACTTCCACGTAGGTCAGGCCGTTGTGGCCACTGATGGTAATATTCAAGGTACTAAGTGGGGAGGTAAATGGCTGGATGCTTACCTACGTGACAGCTTCGTTGCGAAGTCCAAGGCGTGGACTCAGGTGTGGTCTGGTAGGGCTGGCGGTGGGGTAAGTGTGACTGTTTCACAGGATCTCCGCTTCCGCAATATCTgg                                                                                                                                                                                                                                                   <  1:464048/282‑1 (MQ=255)`  
                                                            `ttGGTAGAGGGTCAGATAACAACAATGACTGTACATTCCACTCCTATGTACATGGTACGACCTTAACACTCAAGCAGGACTATGCAGTAGTTAACAAACACTTCAACGTAGGTCAGGCCGTTGTGGCCACTGATGGTAATATTCAAGGTACTAAGTGGGGAGGTAAATGGCTGGATGCTTACCTACGTGACAGCTTCGTTGCGAAGTCCAAGGCGTGGACTCAGGTGTGTTCTGGTAGGGCTGGCTGTGGGGTAAGTGTGACTGTTTCACAGGAGCCCGGCTTCCGCACTATCTGGATTaa                                                                                                                                                                                                                                              >  1:109977/1‑301 (MQ=255)`  
                                                               `gTAGAGGGTCAGATAACAACAATGACTGTACCTTCCACGCCTATGTACATGGTACGACCTTAACACTCAAGCAGGACTAGGCAGTAGTTAACAAACACTTCCACGTAGGTCAGGCCGTTGTGGCCACTGATGGTAATATTCAAGGTACTAAGTGGGGAGGTAAATGGCTGGATGCTTACCTACGTGACAGCTTCGTTGCGAAGTCCAAGGCGTGGACTCAGGTGTGGTCTGGTAGGTCTGGCGGTGGGGTAAGTGTGACTTTTTCACAGGATCTCCGCTTCCGCAATATCTGGATTAAgtg                                                                                                                                                                                                                                           <  1:52647/301‑1 (MQ=255)`  
                                                                  `gagGGTCAGATAACAACAATGACTGTACCTTCCACTCCTATGTACATGGTACGACCTTAACACTCAAGCAGGACTATGCAGTAGTTAACAAACACTTCCACGTAGGTCAGGCCGTTGTGGCCAATGATGGTAATATTCAAGGTACTAAGTGGGGAGGTAAATGGCTGGATGCGTACCTACGTGACAGCTTCGTTGCGAAGTCCAAGGCGTGGGCTCAGGTGTGGTCTGGTAGGGCTGGCGGTGGGGTAAGTGTGACTGTTTCACATGATCTCAGCTTCCGCAATATCTGGATTAAGTGTGc                                                                                                                                                                                                                                        >  1:158007/1‑301 (MQ=255)`  
                                                                  `gagGGTCAGATAAAAACAATGACTGTACCTTCCACTCCTATGTACATGGTACGACCTTAACACTAAAGCAGGACTATGCAGTAGTTAACAAACACTTCCACGTAGGTCAGGCCGTTGTGGCCACTGATGGTAATATTAAAGGTACTAAGTGGGGAGGTAAATGGCTGGATGCTAAAATATGTGACAGCTTCGTTGCGAAGTCCAAGGCGTGGACTCAGGTGTGGTCAGGTATGGCTGGCGTTGGGGTACGTGTGACTGTTTCACAGGATCTCCGCTTCCGCACTATCTGGTTTATGTGGGc                                                                                                                                                                                                                                        >  1:281704/1‑301 (MQ=255)`  
                                                                     `ggTCAGATAACAACAATGACTGTACCTTCCACTCCTATGTACATGGTACGACCTTAACACTCAAGCAGGACTATGCAGTAGTTAACAAACACTTCCACGTAGGTCAGGCCGTTGTGGCCACTGATGGTAATATTCAAGGTACTAAGTGGGGAGGTAAATGGCTGGATGCTTACCTACGTGACAGCTTCGTTGCGAAGTCCAAGGCGTGGACTCAGGTGTGGTCTGGTAGGGCTGGCGGTGGGGTAAGTGTGACTGTTTCACAGGATCTCCGCTTCCGCAATATCTGGATTAAgtgctgtct                                                                                                                                                                                                                                     >  1:277393/1‑295 (MQ=255)`  
                                                                          `gATAACAACAATGACTGTACCTTCCACTCCTATGTACATGGTACGACCTTAACACTCAAGCAGGACTATGCAGTAGTTAACAAACACTTCCACGTAGGTCAGGCCGTTGTAGCCACTGATGGTAATATTCAAGGTACTAAGTGGGGAGGTAAATGGCTGGATGCTTACCTACGTGACAGCTTCGTTGCGAAGTCCAAGGCGTGGACTGAGGTGTGGTCTGGTAGGGCTGGCGGTGGGGTAAGTGTGACTGTTTCACAGGATCTCCGCTTCCGCAATATCTGGATTAAGTGTGCCAACCAct                                                                                                                                                                                                                                >  1:105705/1‑301 (MQ=255)`  
                                                                                    `aTGACTGTACCTTCCACTCCTATGTACATGGTACGACCTTAACACTAAAGCAGGACTATGCAGTAGTTAACAAACACTTCCACGTAGGTCAGGCCGTTGTGGCCACTGATGGTAATATTCAAGGTACTAAGTGGGGAGGTAAATGGCTGGATGCTTACCTACGTGACAGCTTCGTTGCGAAGTCCAAGGCGTGGACTCAGGTGTGGTCTGGTAGGGCTGGCGGTGGGGTAAGTGTGACTGTTTCACAGGATCTCCGCTTCCGCAATATCTGGATTAAGTGTGCCAACAACTCTTGGAActt                                                                                                                                                                                                                      >  1:152564/1‑301 (MQ=255)`  
                                                                                     `tGACTGTACCTTCCACTCCTATGTACATGGTACGACCTTAACACTCAAGCAGGACTATGCAGTAGTTAACAAACACTTCCACGTAGGTCAGGCCGTTGTGGCCACTGATGGTAATATTCAAGGTACTAGGTGGGGAGGAAAATGGCTGGATGCTTACCTACGTGACAGCTTCGTTGCGAAGTCCAAGGCGTGGACTCAGGTGTGGTCTGGTAGGGCTGGCGGTGGGTTAGTGGTGAGTGTTTACCAGGATCTCCGCTTCCGCACTCTCTGGTTTAGTTGTGCCAAGACCTCTTGGAActtc                                                                                                                                                                                                                     >  1:485532/1‑301 (MQ=255)`  
                                                                                     `tGACTGTACCTTCCACTCCTATGTACATGGTACGACCTTAACACTCAAGCAGGACTATGCAGTAGTTAACAAACACTTCCACGTAGGTCAGGCCGTTGTGGCCACTGATGGTAATATTCAAGGTACTAAGTGGGGAGGTAAATGGCTGGATGCTTACCTACGTGACAGCTTCGTTGCGAAGTCCAAGGCGTGGCCTCAGTTGTGGTCTGGTAGGGCTGGCGGGGGGGTAAGTGTGACTGTTTCACAGGAGCCTCGCTTCCGCAATCTCTGGATTAAGTGTGCCAACAACACTTGGAActtc                                                                                                                                                                                                                     >  1:333967/1‑301 (MQ=255)`  
                                                                                     `tGACTGTACCTTCCACTCCTATGTACATGGTACGACCTTAACACTCAAGCAGGACTATGCAGTAGTTAACAAACACTTCCACGTAGGTCAGGCCGTTGTGGCCACTGATGGTAATATTCAAGGTACTAAGTGGGGAGGTAAATGGCTGGATGCTTACCTACGCGACAGCTTCGTTGCGACGTCCAAGGCGTGGACTCAGGTGTTGTCTGGTAGGGCTGGCGGTTGGGTAAGTGTGACTGTTTCACAGGATCTCCGCTTCCGCAATAGCTGGATTAAGTGTGCCAACAACTCTTGGAActtt                                                                                                                                                                                                                     >  1:56773/1‑300 (MQ=255)`  
                                                                                     `tGACTGTACCTTCCACTCCTATGTACATGGTACGACCTTAACACTCAAGCAGGACTATGCAGTAGTTAACAAACACTTCCACGTAGGTCAGGCCGTTGTGGCCACTGATGGTAAAATTCAAGGTACTAAGTGGGGAGGTAAATGGCTGGATGCTTACCTACGTGACAGCTTCGTTGCGAAGTCCAAGGCGTGGACTCAGGTGTGGTCTGGTAGGGCTGGGGGTGGGGTAAGGTTGACTGTTTGACAGGATCTCCGCTTCCGCAATATCTGGATTAAGTGTGCCAACAACTCTTGGAActtc                                                                                                                                                                                                                     >  1:324603/1‑301 (MQ=255)`  
                                                                                        `cTGTACCTTCCACTCCTATGTACATGGTACGACCTTAACACTCAAGCAGGACTATGCAGTAGTTAACAAACACTTCCACGTAGGTCAGGCCGTTGTGGCCACTGATGGTAATATTCAATGTACTAAGTGGGGAGGTAAATGGCTGGATGCTTACCTACGTGACAGCTTCGTTGCGAAGTCCAAGGCGTGGACTCAGGTGTGGTCTGGTAGGGCTGTCGGTGGGGTCAGTGTGACTGTTTCACAGGATCTCCGATTCCGCAATATCTGGATTAAGTGTGCcatcttaatccagatattccgg                                                                                                                                                                                                                  >  1:370140/1‑281 (MQ=255)`  
                                                                                        `cTGTACCTTCCACTCCTATGTACATGGTACGACCTTAACACTCAAGAAGGACTATGCAGTAGTTAACAAACACTACCACGTAGGTAAGGCCGTTGTGGCCACTGATGGTAATATTCAAGGTACTAAGTGGGGAGGTAAATGGCTGGATGCTTACATACGGGACAGCTTCGTTGCGAAGTCCAAGGCGTGGACTCAGGTGTGGTCTGGTAGGGCAGGCGGTGGGGTAAGTGTGACTGTTTCACAGGAACTCCGCTTTCGCAATATCCGGATGAAATGTGCCAACCACTCCTGGAActcattc                                                                                                                                                                                                                  >  1:45125/1‑296 (MQ=255)`  
                                                                                            `aCCTTCCACTCCTATGTACATGGTACGACCTTAACACTCAAGCAGGACTATGCAGTAGTTAACAAACACTTCCACGTAGGTCAGGCCGTTGTGGCCACTGATGGTAATATTCAAGGTACTAAGTGGGGAGGTAAATGGCTGGATGCTTACCTGCGTGACAGCTTCGTTGCGAAGTCCAAGGCGTGGACTCAGGTGTGGTCTGGTAGGGCTGGCGGTGGGGTAAGTGTGACTGTTTCACAGGATCTCCGCTTCCGCAATATCTGGATTAAGTGTGCCAACACCTATTGGAACTTCTTCCGTa                                                                                                                                                                                                              >  1:361587/1‑301 (MQ=255)`  
                                                                                                      `ccTATGTACATGGTACGACCTTAACACTCAAGCAGGACTATGCAGTAGTTAACAAACACTTCCACGTAGGTCAGGCCGTTGTGGCCACTGATGGTAATATTCAAGGTACTAAGTGGGGAGGTAAATGGCTGGATGCTTACCTACGTGACAGCTTCGTTGCGAAGTCCAAGGCGTGGACTCAGGTGTGGTCTGGTAGGGCTGGCGGTGGGGTAAGTGTGACTGTTTCACAGGATCTCCGCTTCCGCAATATCTGGATTAAGTGTGCCAACAACTCTTGGAACTTCTTCCGTAcctgtctctt                                                                                                                                                                                                    >  1:472545/1‑292 (MQ=255)`  
                                                                                                      `ccTATGTACATGGTACGACCTTAACACTCAAGCAGGACTATGCAGTAGTTAACAAACACTTCCACGTAGGTCAGGCCGTTGTGGCCACTGATGGTAATATTCAAGGTACTAAGTGGGGAGGTAAATGGCTGGATGCTTACCTACGTGACAGCTTCGTTGCGAAGTCCAAGGCGTGGACTCAGGTGTGGTCTGGTAGGGCTGGCGGTGGGGTAAGTGTGACTGTTTCACAGGATCTCCGCTTCCGCAATATCTGGATTAAGTGTGCCAACAACTCTTGGAACTTCTTCCGTAATGGCCCCGa                                                                                                                                                                                                    >  1:175725/1‑301 (MQ=255)`  
                                                                                                      `ccTATGTACATGGTACGACCTTAACACTCAAGCAGGACTATGCAGTAGTTAACAAACACTTCCACGTAGGTCAGGCCGTTGTGGCCACTGATGGTAATATTCAAGGTACTAAGTGGGGAGGTAAATGGCTGGATGCTTACCTACGTGACAGCTTCGTTGCGAAGTCCAAGGCGTGGACTCAGGTGTGGTCTGGTAGGGCTGGCGGTGGGGTAAGTGTGACTGTTTCACAGGATCTCCGATTCCGCAATATCTGGATTAAGTGTGCCAACAACTCTTGGAACTTCTTCCGTACTGGCCCCGa                                                                                                                                                                                                    >  1:7954/1‑301 (MQ=255)`  
                                                                                                      `ccTATGTACATGGTACGACCTTAACACTCAAGCAGGACTATGCAGTAGTTAACAAACACTTCCACGTAGGTCAGGCCGTTGTGGCCACTGATAGTAATATTCAAGGTACTAAGTGGGGAGGTAAATGGCTGGATGCTTACCTACGTGACAGCTTCGTTGCGAAGTCCAAGGCGTGGACTCAGGTGTGGTCTGGTAGGGCTGGCGGTGGGGTAAGTGTGACTGTTTCACAGGCACTCCGCTTCCGCAATATCTGGATTAAGTGTTCCAACAACTCTTGGCACTTCTTCCGTCCTGTCCCCGa                                                                                                                                                                                                    >  1:95796/1‑301 (MQ=255)`  
                                                                                                        `tATGTACATGGTACGACCTTAACACTCAAGCAGGACTATGCAGTAGTTAACAAACACTTCCACGTAGGTCAGGCCGTTGTGGCCACTGATGGTAATATTCAAGGTACTAAGTGGGGAGGTAAATGGCTGGATGCTTACCTACGTGACAGCTTCGTTGCGAAGTCCAAGGCGTGGACTCAGGTGTGGTCTGGTAGGGCTGGCGGTGGGGTAAGTGTGACTGTTTCACAGGATCTCCGCTTCCGCAATATCTGGATTAAGTGTGCCAACAACTCTTGGAACTTCTTCCGTACTGGCCCCGATg                                                                                                                                                                                                  >  1:199609/1‑301 (MQ=255)`  
                                                                                                         `aagagacaggTACGACCTTAACACTCAAGCAGGACTATGCAGTAGTTAACAAACACTTCCACGTAGGTCAGGCCGTTGTGGCCACTGATGGTAATATTCAAGGTACTAAGTGGGGAGGTAAATGGCTGGATGCTTACCTACGTGACAGCTTCGTTGCGAAGTCCAAGGCGTGGACTCAGGTGTGGTCTGGTAGGGCTGGCGGTGGGGTAAGTGTGACTGTTTCACAGGATCTCCGCTTCCGCAATATCTGGATTAAGTGTGCCAACAACTCTTGGAACTTCTTCCGTACTGGCCCCGATgg                                                                                                                                                                                                 <  1:319639/293‑1 (MQ=255)`  
                                                                                                         `aTGTACATGGTACGACCTTAACACTCAAGCAGGACTATGCAGTAGTTAACAAACACTTCCACGTAGGTCAGGCCGTTGTGGCCACTGATGGTAATATTCAAGGTACTAAGTGGGGAGGTAAATGGCTGGATGCTTACCTACGTGACAGCTTCGTTGCGAAGTCCAAGGCGTGGACTCAGGTGTGGTCTGGTAGGGCTGGCGGTGGGGTAAGTGTGACTGTTTCACAGGATCTCCGCTTCCGCAATATCTGGATTAAGTGTGCCAACAACTCTTGGAACTTCTTCCGTACTGGCCCCGATgg                                                                                                                                                                                                 >  1:87266/1‑301 (MQ=255)`  
                                                                                                             `aCATGGTACGACCTTAACACTCAAGCAGGACTATGCAGTAGTTAACAAACACTTCCACGTAGGTCAGGCCGTTGTGGCCACTGATGGTAATATTCAAGGTACTAAGTGGGGAGGTCAATGGCTGGATGCTTACCTACGTGACAGCTTCGTTGCTAAGTCCAAGGCGTGGACTCAGGTGTGGTCTGGTTGGGCTGGCGGTGGGGTAAGTGTGCATATTTCAACGGCTCTCCGCTTCCGCAATATTTGAATTACTTGTGCCACACACACTTGGAACTTCTTCCGTCCTGGTCTCGAAGGAATc                                                                                                                                                                                             >  1:292754/1‑301 (MQ=255)`  
                                                                                                             `aCATGGTACGACCTTAACACTCAAGCAGGACTATGCAGTAGTTAACAAACACTTCCACGTAGGTCAGGCAGTTGTGGCCACTGATGGTAATATTCAAGGTACTAAGAGGGGAGGTAAATGGCTGGATGCTTACCTAAGTGACAGCTTCGTTGCGAAGTCCAAGGCGTGGACTCAGGTTTGGTCTGGTAGGGCTGGCGGTGGGGTATGTGTGACTGTTTCAACGGATCTCTGCTTACGCAATATCTGGATTAAGTGTGACAACAACTATTGGACCTTCTTCCGTACTGGccacactagaatt                                                                                                                                                                                             >  1:125325/1‑290 (MQ=255)`  
                                                                                                                 `ggTACGACCTTAACACTCAAGCAGGACTATGCAGTAGTTAACAAACACTTCCACGTAGGTCAGGCCGTTGTGGCCACTGATGGTAATATTCAAGGTACTAAGTGTGGAGGTAAATTGCTGGATGCTTACCTACGTGACAGCTTCGTTGCGAAGTCCAAGGCGTGGACTCAGGTGTGGTCTGGTAGGGCTGGAGGTGGGGTAAGTGTGACTGTTTCACAGGATTTCCGCTTCCGCAATATCTGGATTAAGTGTGACAACACCTCTTGGAACTTCTTCCGTACTGGCCCCGATGGACTccctt                                                                                                                                                                                         >  1:507964/1‑297 (MQ=255)`  
                                                                                                                  `gTACGACCTTAACACTAAAGCAGGAATATGCAGTAGTTAACAAACACTTCCACGTAGGTCAGGCCGTTGTGGCCACTGATGGTAATATTCAAGGTACTAAGTGGGGAGGTAAATGGCTGGATGCTTACCTACGTGACAGCTTCGTTGCGAAGTCCAAGGCGTGGACTCAGGTGTGGTCTGGTAGGGCTGGCGGTGGGGTAAGTGTGACTGTTTCACAGGATCTCCGCTTCCGCAATATCTGGATTAAGTGTGCCAACAACTCTTGGAACTTCTTCCGTACTGGCCCCGATGGAATCTACtt                                                                                                                                                                                        <  1:447462/301‑1 (MQ=255)`  
                                                                                                                        `ccTTAACACTCAAGCAGGACTATGCAGTAGTTAACAAACACTTCCACGTAGGTCAGGCCGTTGTGGCCACTGATGGTAATATTCAAGGTACTAAGTGGGGAGGTAAATGGCTGGTTGCTTACCTACGTGACAGCTTCGTTGCGAAGTCCAAGGCGTTGACTTAGGTGTGGTCTGGTAGGGCTGGCGGTGGGGTAAGTGTGACTGTTTCACAGGATCTCCGCTTCCGCAATATCTGGATTAAGTGTGCCAACAACTCTTGGAACTTCTTCCGTACTGGCCCCGTTGGAATCTACTTCATGGc                                                                                                                                                                                  >  1:68482/1‑301 (MQ=255)`  
                                                                                                                        `ccTTAACACTCAAGCAGGACTAGTCAGTAGTTTACAAACACTTCCACGTAGGTCAGGCCGTTGTTGCCACTGATGGTAATATTCAAGGTACTAAGTGGGGAGGTAAATGGATGGATGCTTACCTACGTGACAGCTTCGTTGCGAAGTCCAAGGCGTGGACTCAGGTGTGGTCTGGTAGGGCTGGCGGTGTGGTAAGTGTGACTGTTTCACAGGATCTCCGCTTCCGCAATATCTGGATTAAGTGTGCCAACAACTCTTGGAACTTCTTCCGTACTGGCCCCGATGGAATCTACTTCATAGc                                                                                                                                                                                  <  1:240593/301‑1 (MQ=255)`  
                                                                                                                               `acTCAAGCAGGACTATGCAGTAGTTAACAAACACTTCCACGTAGGTCAGGCCGTTGTGGCCACTGATGGTAATATTCAAGGTACTAAGTGGGGAGGTAAATGGCTGGATGCTTACCTACGTGACAGCTTCGTTGCGAAGTACAAGGCGTGGACTCAGGTGTGGTCTGGTAGGGCTGGCGGTGGGGTAAGTGTGACTGTTTCACAGGATCTCCGCTACCGCAATATCTGGATTAAGTGTGCCAACAACTCTTGGAACTTCTTCCGTACTGGCCCCGATGGAATCTACTTCATAGCCTCTGAt                                                                                                                                                                           <  1:83873/301‑1 (MQ=255)`  
                                                                                                                                 `tccaGCATGACTATGCAGTAGTTAACAAACACTTCCACGTAGGTCAAGCCGTTGTGGCCACTGATGGTAATATTCAAGGTACTAAGTTGGGAGGTAAATGGCTGGATGCTTACCTACGTGACAGCTTCGTTGCGAAGTCCAAGGCGTGGACTCAGGTGTGGTCTGGTAGGGCTGGCGGTGGGGTAAGTGTGACTGTTTCACAGGATCTCCGATTCCGCAATATCTGGATTAAGTGTGCCAACAACTCTTGGAACTTCTTCCGTACTGGCCCCGATGGAATCTACTTCATAGCCTCTGAtgg                                                                                                                                                                         <  1:60301/298‑1 (MQ=255)`  
                                                                                                                                  `cAAGCAGGACTATGCAGTAGTTAACAAACACTTCCACGTAGGTCAGGCCGTTGTGGCCACTGATGGTAATATTCAAGGTACTAAGTGGGGAGGTAAATGGCTGGATGCTTACCTACGTGACAGCTTCGTTGCGAAGTCCAAGGCGTGGACTCAGGTGTGGTCTGGTAGGGCTGGCGGTGGGGTAAGTGTGACTGTTTCACAGGATCTCCGCTTCCGCAATATCTGGATTAAGTGTGCCAACAACTCTTGGAACTTCTTCCGTACTGGACCCGATGGAATCTACTTCATAGCCTCTGAtctt                                                                                                                                                                        >  1:288047/1‑298 (MQ=255)`  
                                                                                                                                   `aaGCAGGACTATGCAGTAGTTAACAAACACTTCCACGTAGGTCAGGCCGTTGTGGCCACTGATGGTAATATTCAAGGTACTAAGTGGGGAGGTAAATGGCTGGATGCTTACCTACGTGACAGCTTCGTTGCGAAGTCCAAGGCGTGGACTCAGGTGTGGTCTGGTAGGGCTGGCGGTGGGGTAAGTGTGACTGTTTCACAGGATCTCCGCTTCCGCAATATCTGGATTAAGTGTGCCAACAACTCTTTGAACTTCTTCCGTACTGGCCCCGATGGAATCTACTTCATAGCCTCTGAtggtg                                                                                                                                                                       >  1:442661/1‑301 (MQ=255)`  
                                                                                                                                     `gtataagagacaggtattTAAAAAACAAGTCCACGTAGGTCAGGCCGTTGTGGCCAATGATGGTAATATTCAAGGTACTAAGGGGGGAGGTAAATGGCTGGATGCTTACCTACGTGACAGCTTCGTTGCGAAGTCCAAGGCGTGGACTCAGGTGTGGTCTGGTAAGGCTGGCGGTGGAGTAAGTGTGACTGTTTCACAGGATCTCCGCTTCCGCAATATCTGGATTAAGTGTGCCAACAACTCTTGGAACTTCTTCCGTACTGGCCCCGATGGAATCTACTTCATAGCCTCTGATGGTGGa                                                                                                                                                                     <  1:351455/288‑1 (MQ=255)`  
                                                                                                                                     `gCAGGACTATGCAGTAGTTAACAAACACTTCCACGTAGGTCAGGCCGTGGTGGCCACTGATGGTAATATTCAAGGTACTAAGTGGGGAGGTAAATGGCTGGATGCTTACCTACGTGACAGCTTCGTTGCGAAGTCCAAGGCGTGGACTCAGGTGTGGTCTGGTAGGGCTGGCGGTGGGGTAAGTGTGACTGTTTCACAGGATCTCCGCTTCCGCAATATCTGGATTAAGTGTGCCAACAACTCTTGGAACTTCTTCCGTACTGGCCCCGATGGAATCTACTTCATAGCCTCTGATGGTGGa                                                                                                                                                                     <  1:147624/301‑1 (MQ=255)`  
                                                                                                                                        `ggACTATGCAGTAGTTAACAAACACTTCCACGTAGGTCAGGCCGTTGTGGCCACTGATGGTAATATTCAAGGTACTAAGTGGGGAGGTAAATGGCTGGATGCTTACCTACGTGACAGCTTCGTTGCGAAGTCCAAGGCGTGGACTCAGGTGTGGTCTGGTAGGGCTGGCGGTGGGGTAAGTGTGACTGTTTCACAGGATCTCCGCTTCCGCAATATCTGGATTAAGTGTGCCAACAACTCTTGGAACTTCTTCCGTACTGGCCCCGATGGAATCTACTTCATAGCCTCTGATGGTGGATgg                                                                                                                                                                  >  1:429363/1‑301 (MQ=255)`  
                                                                                                                                           `atATGGAGTAGGTAACAAACACTTGCACGTAGGTCAGGCCGTTTGGGCCAATGATGGTAATATTAAAGGTACTAAGTGGGGAAGTAAATGGCTGGATGCTTACCTACGTGACAGCTTCGTTGCGAAGTACAAGGCGTGGAGGCAGGTGTGGTCTGGTAGGGCTGGCGGTGGGGTAAGTGTTACTGTTTCACAGGATCTCCACTTCCGCAATATCTGGATTAAGTGTGCCAACAACTCTTGGAACTTCTTCCGTACTGGCCCCGATGGAATCTACTTCATAGCCTCTGATGGTGGATGGTTa                                                                                                                                                               <  1:424526/300‑1 (MQ=255)`  
                                                                                                                                             `aTGCAGTAGTTAACAAACACTTCCACGTAGGTCAGGCCGTTGTGGCCACTGATGGTAATATTCAAGGTACTAAGTGGGGAGGTAAATGGCTGGATGCTTACCTACGTGACAGCTTCGTTGCGAAGTCCAAGGCGTGGCCTCAGGTGTGGTCTGGTAGGGCTGGCGGTGGGGTAAGTGTGACTGTTTCACAGGATCTCCGCTTCCGCAATATCTGGATTAAGTGTGCCAACAACTCTTGGAACTTCTTCCGTCCTGGCCCCGATGGAATCTACTTCATAGCCTCTGATGGTGGATGGTTACg                                                                                                                                                             >  1:213080/1‑301 (MQ=255)`  
                                                                                                                                             `aTGCAGTAGTTAACAAACACTTCCACGTAGGTCAGGCCGTTGTGGCCACTGATGGTAATATTCAAGGTACTAAGTGGGGAGGTAAATGGCTGGATGCTTACCTACGTGACAGCTTCGTTGCGAAGTCCAAGGCGTGGACTCAGGTGTGGTCTGGTAGGGCTGGCGGTGGGGTAAGTGTGACTGTTTCACAGGATCTCCGCCTCCGCAATATCTGGATTAAGTGTGCCAACAACCCTTGGAACTTCTTCCGTACTGGCCCCGATGGAATCTACTTCATAGCATCTGATGGTGGATGGTTACg                                                                                                                                                             >  1:33798/1‑301 (MQ=255)`  
                                                                                                                                               `gCAGTAGTTAACAAACACTTCCACGTAGGTCAGGCCGTTGTGGCCACTGATGGTAATATTCAAGGTACTAAGTGGGGAGGTAAATGGCTGGATGCTTACCTACGTGACAGCTTCGTTGCGAAGTCCAAGGCGTGGACTCAGGTGTGGTCTGGTAGGGCTGGCGGTGGGGTAAGTGTGACTGTTTCACAGGATCTCCGCTTCCGCAATATCTGGATTAAGTGTGCCAACAACTCTTGGAACTTCTTCCGTACTGGCCCCGATGGAATCTACTTCATAGCCTCTGATGGTGGATGGTTACGAt                                                                                                                                                           <  1:460623/301‑1 (MQ=255)`  
                                                                                                                                                 `agtagtTAACAAACACTTCCACGTAGGTCAGGCCGTTGTGGCCACTGATGGTAATATTCAAGGTACTAAGTGGGGAGGTAAATGGCTGGATGCTTACCTACGTGACAGCTTCGTTGCGAAGTCCAAGGCGTTGACTCAGGTGTGGTCTGGTAGGGCTGGCGGTGGGGTAAGTGTGACTGTTTCACAGGATCTCCGCTTCCGCAATATCTGGATTAAGTGTGCCAACAACTCTTGGAACTTCTTCCGTACTGGCCCCGATGGAATCTACTTCATAGCATCTGATGGTGGATGGTTACGATTc                                                                                                                                                         <  1:204254/301‑1 (MQ=255)`  
                                                                                                                                                  `gtagtTAACAAACACTTCCACGTAGGTCAGGCCGTTGTGGCCATTGATGGTAATATTCAAGGTACTAAGTGGGGAGGTAAATGGCTGGATGCTTACCTACGTGACAGCTTCGTTGCGAAGTCCAAGACGTGGACTCAGGTGTGGTCTGGTAGTGCTGGCGGTGGGGTAAGTGTGACTGCTTCACAGGATCTCCGCTTCCGCAATATCTGGATTAAGTGTGCCTACAACTCTTGGAACTTCGTCCGTACTGGCACCGATGGAAGCTACTTCATAGCCTCGGATGGTGGATGGTTACGATTcc                                                                                                                                                        >  1:64664/1‑301 (MQ=255)`  
                                                                                                                                                  `gtagtTAACAAACACTTCCACGTAGGTCAGGCCGTTGTGGCCACTGATGGTAATATTCAAGGTACTAAGTGGGGAGGTAAATGGCTGGATGCTTACCTACGTGACAGCTTCGTTGCGAAGTCCAAGGCGTGGACTCAGGTGTGGTCTGGTAGGGCTGGCGGTGGGGTAAGTGTGACTGTTTCACAGGAGCTCCGCTTCCGCAATATCTGGATTAAGTGTGCCAACAACGCTGGGAACTTCTTCCGTACTGGCACAGATGGAATCTACTTCATAGCCTCTGATGGTGGATGGTTACGATTca                                                                                                                                                        >  1:142312/1‑300 (MQ=255)`  
                                                                                                                                                     `gtTAACAAACACTTCCACGTAGGTCAGGCCGTTGTGGCCACTGATGGTAATATTCAAGGTACTAAGTGGGGAGGTAAATGGCTGGATGCTTACCTACGTGACAGCTTCGTTGCGAAGTCCAATGCGTGGACTCAGGTGTGGTCTGGTAGGGCTGGCGGTGGGGTAAGTGTGACTGTTTCACAGGATCTCCGCTTCCGCAATATCTGGATTAAGTGTGCCAACAACTCTTGGAACTTCTTCCGTACTGGCTCCGATGGAATCTACTTCATAGCCTCTGATGGTGGATGGTTACGATTCCaaa                                                                                                                                                     >  1:73787/1‑301 (MQ=255)`  
                                                                                                                                                       `tAACAAACACTTCCACGTAGGTCAGGCCGTTGTGGCCACTGATGGTAATATTCAAGGTACTAAGTGGGGAGGTAAATGGCTGGATGCTTACCTACGTGACAGATTCGTTGCGAAGTCCAAGGCGTGGACTCAGGTGTGGTCTGGTAGGGCTGGCGGTGGGGTAAGTGTGACTGTTTTACAGGATCTCAGCTTCCGCAATATCTGGATTAAGTGTGCCAACAACTCTTGGAACTTCTTCCGTACTGGCACCGATGGAATCTACTTCATAGCATCTGATGGTGGATGGTTACGATTCCAAATa                                                                                                                                                   >  1:401278/1‑301 (MQ=255)`  
                                                                                                                                                        `aacaaacaCTTCCACGTAGGTCAGGCCGTTGTGGCCACTGATGGTAATATTCAAGGTACTAAGTGGGGAGGTAAATGGCTGGATGCTTACCTACGTGACAGCTTCGTGGCGAAGTCCAAGGCGTGGACTCAGGTGTGGTCTGGTAGGGCTGGCGGTGGGGTAAGTGTGACTGTTTCACAGGATCTCCGCTTCCGCAATATCTGGATTAAGTGTGCCAACAACTCTTGGAACTTCTTCCGTACTGGCCCCGATGGAATCTACTTCATAGCCTCTGATGGTGGATGGTTACGATTCCAAATac                                                                                                                                                  <  1:480675/301‑1 (MQ=255)`  
                                                                                                                                                                     `aCGTAGGTCAGGCCGTTGTGGCCACTGATGGTAATATTCAAGGTACTAAGTGGGGAGGTAAATGGCTGGATGCTTACCTACGTGACAGCTTCGTTGCGAAGTCCAAGGCGTGGACTCAGGTGTGGTCTGGTAGGGCTGGCGGTGGGGTAAGTGTGACTGTTTCACAGGATCTCCGCTTCCGCAATATCTGGATTAAGTGTGCCAACAACTCTTGGAACTTCTTCCGTACTGGCCCCGATGGAATCTACTTCATAGCCTCTGATGGTGGATGGTTACGATTCCAAATACACTCCAACGGtct                                                                                                                                     >  1:420481/1‑301 (MQ=255)`  
                                                                                                                                                                          `tgtataagagacagggCCACTGATGGTAATATTCAATGTACTAAGTGGGGGGGGAAATGGCTGGATGCTTCCCTAAGTGACAGCTTCGTTGCGAAGTCCAAGGCTAGGACTCAGGTGTGTTCTGGTAGGGTTGGCGGTGGGGTAAGTGTGACTATTTCACAGGATCTCCGATTCCGCAATATCTGGATTAAGTGTGCCAACAACTCTGGGAACTTCTTCCGTACTGGTCCCGATGGAATCTACTTCATAGCCTCTGATGGTGGATGGTTACGATTCCAAATACACTCCAACGGTCTCGGAt                                                                                                                                <  1:255162/287‑1 (MQ=255)`  
                                                                                                                                                                          `ggTCAGGCCGTTGTGGCCACTGATGGTAATATTCAAGGTACTAAGTGGGGAGGTAAATGGCTGGATGCTTACCTACGTGACAGCTTCGTTGCGAAGTCCAAGGCGTGGACTCAGGTGTGGTCTGGTAGGGCTGGCGGTGGGGTAAGTGTGACTGTTTCACAGGATCTCCGCTTCCGCAATATCTGGATTAAGTGTGCCAACAACTCTTGGAACTTCTTCCGTACTGGCCCCGATGGAATCTACTTCATAGCCTCTGATGGTGGATGGTTACGATTCCAAATACACTCCAACGGTCTCGGAt                                                                                                                                >  1:309025/1‑301 (MQ=255)`  
                                                                                                                                                                          `ggTCAGGCCGTTGTGGCCACTGATGGTAATATTCAAGGTACTAAGTGGGGAGGTAAATGGCTGGATGCTTACCTACGTGACAGCTTCGTTGCGAAGTCCAAGGCGTGGACTCAGGTGTGGTCTGGTAGGGCTGGCGGTGGGGTAAGTGTGACTGTTTCACAGGATCTCCGCTTCCGCAATATCTGGATTAAGTGTGCCAACAACTCTTGGAACTTCCTCCGTACTGGCCCCGATGGAATCTACTTCATAGCCTCTGATGGTGGATGGTTACGATTCCAAATACACTCCAACGGTCTCGGAt                                                                                                                                <  1:167592/301‑1 (MQ=255)`  
                                                                                                                                                                              `aGGCCGTTGTGGCCACTGATGGTAATATTCAAGGTACTAAGTGGGGAGGTAAATGGCTGGATGCTTACCTACGTGTCAGCTTCGTTGCGAAGTCCAAGGCGTGGACTCAGGTGTGGTCTGGTAGGGCTGGCGGTGGGGTAAGTGTGACTGTTTCACAGGATCTCCGCTTCCGCAATATCTGGCTTAAGTGTGCCAACAACTCTTGGAACTTCTTCCGTACTGGCCCCGATGGAATCTACTTCATAGCCTCTGATGGTGGATGGTTACGTTTCCAAATACACTCCAACGGTCTCGGATTCaa                                                                                                                            >  1:250425/1‑301 (MQ=255)`  
                                                                                                                                                                                 `ccGATGTGGCTACTGAGGGTAATATTCAAGGTACTAAGTGGGTAGGTAAATGGCTGGATGCTTACCTACGTGATAGCTTCGTTGCGAAGTCCAAGGCGTGGACTCAGGTGTGGTCTGGTAGGGCTGGCGGTGGGGTAAGTGTGACTGTTTCACAGGATCTCCGCTTCCGAAATATCTGGATTAAGTGTGCCAACAACTCTTGGAACTTCTTCCGTACTGGCCCCGATGGAATCTACTTCATAGCCTCTGATGGTGGATGGTTACGATTCCAAATACACTCCAACGGTCTCGGATTCAAGaa                                                                                                                         <  1:306014/301‑1 (MQ=255)`  
                                                                                                                                                                                    `tggtgGCCACTGATGGTAATATTCAAGGTACGAAGGGGGGAGGTAAATGGCTGGATGCTTACCTACGTGAAAGCTTCGTTGCGAAGTCCAATGCGTGGACTCAGGTGTGGTCTGGTAGGGCTGGCGGTGGGGTAAGTGTGACTGTTTCACAGGATCTCCGCTTCCGCAATATCTGGATTAAGTGTGCCAACAACTCTTGGAACTTCTGCCGTACTGGCCCCGATGGAATATACTTCATAGCATCTGATGGTGGATGGTTACGATTCCAAATACACTCCAACGGTCTCGGATTCAAGAatat                                                                                                                      <  1:114418/299‑1 (MQ=255)`  
                                                                                                                                                                                         `aagagacagggTAATATTCAAGGTACTAAGTGGGGAGGTAAATGGCTGGATGCTTACCTACGTGACAGCTTCGTTGCGAAGTCCAAGGCGTGGACTCAGGTGTGGTCTGGTAGGGCTGGCGGTGGGGTAAGTGTGACTGTTTCACAGGATCTCCGCTTCCGCAATATCTGGATTAAGTGTGCCAACAACTCTTGGAACTTCTTCCGTACTGGCCCCGATGGAATCTACTTCATAGCCTCTGATGGTGGATGGTTACGATTCCAAATACACTCCAACGGTCTCGGATTCAAGAATATTGCAg                                                                                                                 <  1:26795/292‑1 (MQ=255)`  
                                                                                                                                                                                           `cACTGATGGTAATATTCAAGGTACTAAGTGGGTAGGTAAATGGCTGGATGCTTACCTACGTGGCAGCTTCGTTGCGAAGTCCAAGGCGTGGACTCAGGTGTGGTCTGGTAGGGCTGGCGGTGGGGTAAGTGTGACTGTTTCACAGGATCTCCGCTTCCGCAATATCTGGATTAAGTGTGCCAACAACTCTTGGAACTTCTTCCGTACTGGCCCCGATGGAATCTACTTCATAGCCTCTGATGGTGGATGGTTACGATTCCAAATACACTCCAACGGTCTCGGATTCAAGAATATTGCAGAc                                                                                                               <  1:296777/301‑1 (MQ=255)`  
                                                                                                                                                                                               `gATGGTAATATTCAAGGTACTAAGTGGGGAGGTAAATGGCTGGATGCTTACCTACGTGACAGCTTCGTTGCGAAGTCCAAGGCGTTGACTCAGGTGTGGTCTGGTAGGGCTGGCGGTGGGGTAAGTGTGACTGTTTCACAGGATCTCCGCTTCCGCAATATCTGGATTAAGTGTGCCAACAACTCTTGGAACTTCTTCCGTACTGGCCCCGATGGAATCTACTTCATAGCCTCTGATGGTGGATGGTTACGATTCCAAATACACTCCAACGGTCTCGGATTCAAGAATATTGCATACAGTc                                                                                                           >  1:302113/1‑301 (MQ=255)`  
                                                                                                                                                                                                   `tataagagacaGGTACTATGTGGGGCGGTTACTGGCTGTATGCTTACCTACGTGACAGCTTCGTTGCGAAGTCCCAGGCGTGGACTCAGGTGTTGTCTGGTAGGGCTGGCGGTGGGGTAAGTGTGACTGTTTCACAGGATCTCCGCTTCCGCAATATCTGGATTAAGTGTGCCAACAACTCTTGGAACTTCTTCCGTACTGGCCCCGATGGAATCTACTTCATAGCCTCTGATGGTGGATGGTTACGATTCCAAATACACTCCAACGGTCTCGGATTCAAGAATATTGCAGACAGTCGTTc                                                                                                       <  1:2598/291‑1 (MQ=255)`  
                                                                                                                                                                                                       `tatTCAAGGTACTAAGTGGGGAGGTAAATGGCTGGATGCTTACCTACGTGACAGCTTCGTTGCGAAGTCCAAGGCGTGGACTCAGGTGTGGTCTGGTAGGGCTGGCGGTGGGGTAAGTGTGACTGTTTCACAGGATCTCCGCTTCCGCAATATCTGGATTAAGTGTGCCCACAACTCTTGGAACTTCTTCCGTACTGGCCCCGATGGAATCTACTTCATAGCCTCTGATGGTGGATGGTTACGATTCCAAATACCCTCCCACGGTCTCGGTTTCAAGAATATTGCCGACAGTCGTTCAGTa                                                                                                   >  1:231708/1‑301 (MQ=255)`  
                                                                                                                                                                                                       `tatTCAAGGTACTAAGTGGGGAGGTAAATGGCTGGATGCTTACCTACGTGACAGCTTCGTTGCGAAGTCCAAGGCGTGGACTCAGGTGTGGTCTGGTAGGGCTGGCGGTGGGGTAAGTGTGACTGTTTCACAGGATCTCCGCTTCCGCAATATCTGGATTAAGTGTGCCAACAACTCTTGGAACTTCTTCCGTACTGGCCCCGATGGAATCTACTTCATAGCCTCTGATGGTGGATGGTTACGATTCCAAATACACTCAAACGGTCTCGGATTCAAGAATATTGCAGACAGTCGTTCAGTa                                                                                                   >  1:494475/1‑301 (MQ=255)`  
                                                                                                                                                                                                             `aGGTACTAAGTGGGGAGGTAAATGGCTGGATGCTTACCTACGTGACAGCTTCGGTGCGAAGTCCAAGGCGTGGACTCAGGTGTGGTCTGGTAGGGCTGGCGGTGGGGTAAGTGTGACTGTTTCACAGGATCTCCGCTTCCGCAATATCTGGATTAAGTGTGCCAACAACTCTTGGAACTTCTTCCGTACTGGCCCCGATGGAATCTACTTCATAGCCTCTGATGGTGGATGGTTACGATTCCAAATACACTCCAACGGTCTCGGATTCAAGAATATTGCAGACAGTCGTTCAGTACCTAAt                                                                                             <  1:235957/301‑1 (MQ=255)`  
                                                                                                                                                                                                              `ggTACTAAGTGGGGAGGTAAATGGCTGGATGCTTACCTACGTTACAGCTTCGTTGCGAAGTCCAAGGCGTGGACTCAGGTGTGGTCTGGTAGGGCTGGCGGTGGGGTAAGTGTGACTGTTTCACAGGCTCTCCGCTTCCGCAATATCTGGATTAAGTGTGCCAACAACTCTTGGAACTTCTTACGTACTTGCCCCTATGGAATCTACTTCATAGCCTCTGATGGTGGATGGTTACGATTCCAAATACACTCCAACGGTCTCGGATTCAAGAATATTGCAGACAGTCGTTCAGTACCTAATg                                                                                            <  1:406670/301‑1 (MQ=255)`  
                                                                                                                                                                                                              `ggTACTAAGTGGGGAGGTAAATGGCTGGATGCTTACCTACGTGACAGCTTCGTTGCGAAGTCCAAGGCGTGGACTCAGGTGTGGTCTGGTAGGGCTGGCGGTGGGGTAAGTGTGACTGTTTCACAGGATCTCCGCTTCCGCAATATCTGGATTAAGTGTGCCAACAACTCTTGGAACTTCTTCCGTACTGGCCCCGATGGAATCTACTTCATAGCCTCTGCTGGTGGATGGTTACGATTCCAAATACACTCCAACGTCCTCGGATTCAAGAATATTGCAGACAGTCGTCCAGTAccctgtc                                                                                            >  1:365544/1‑296 (MQ=255)`  
                                                                                                                                                                                                              `ggTACTAAGTGGGGAGGT‑AATGGCTGGATGCTTACCTACGTGACAGCTTCGTTGCGAAGTCCAAGGCGTGGACTCAGGTGTGGTCTGGTAGGGCTGGCGGTGGGGTAAGTGTGACTGTTTCACAGGATCTCCGCTTCCGCAATATCTGGATTAAGTGTGCCAACAACTCTTGGAACTTCTTCCGTACTGGCCCCGATGGAATCTACTTCATAGCCTCTGATGGTGGATGGTTACGATTCCAAATACACTCCAACGGTCTCGGATTCAAGAATATTGCAGACAGTCGTTcctgtctcttata                                                                                           >  1:476471/1‑289 (MQ=255)`  
                                                                                                                                                                                                               `gTACTAAGTGGGGAGTTAAAGGGCGTGATGCTTACCTACGTGACAGCTTCGGTGCGAAGTCCAAGGAGTGGACTCAGGTGTGGTCTGGTAGGGCTGGCGGTGGGGTAAGTGTGACTGTTTCACAGGATCTCCGCTTCCGCAATATCTGGATTAAGTGTGCCAACAACTCTTGGAACTTCTTCCGTACTGGCCCCGATGGAATATACTTCATAGCCTCTGATGGTGGATGGTTACGATTCCAAATACACTCCAACGGTCTCGGATTCAAGAATATTGCAGACAGTAGTTCAGTACCTAATGc                                                                                           <  1:455532/301‑1 (MQ=255)`  
                                                                                                                                                                                                               `gTACTAAGTGGGGAGGTAAATGGCTGGATGCTTACCTACGTGACAGCTTCGTTGCGAAGTCCAAGGCGTGGACTCAGGTGTGGTCTGGTAGGGCTGGCGGTGGGGTAAGTGTGACTGTTTCACAGGATCTCCGCTTCCGCAATATCTGGATTAAGTGTGCCAACAACTCTTGGAACTTCTTCCGTACTGGCCCCGATGGAATCTACTTCATAGCCTCTGATGGTGGATGGTTACGATTCCAAATACACTCCAACGGTCTCGGATTCAAGAATATTGCAGACAGTCGTTCAGTACCTAATGc                                                                                           >  1:220941/1‑301 (MQ=255)`  
                                                                                                                                                                                                                  `cTAAGTGTGGAGGTAAATGGCTGGATGCTTACCTACGTGACAGCTTCGTTGCGAAGTCCAAGGCGTGGACTCAGGTGTGGTCTGGTAGGGCTGGCGGTGGGGTAAGTGTGACTGTTTCACAGGATCTCCGCTTCCGCAATATCTGGATTAAGTGTGCCAACAACTCTTGGAACTTCTTCCGTACTGGCCCCGATGGAATCTACTTCATAGCCTCTGATGGTGGATGGTTACGATTCCAAATACACTCCAACGGTCTCGGATTCAAGAATATTTCAGACactgtctcttatacacatctccg                                                                                        >  1:19403/1‑279 (MQ=255)`  
                                                                                                                                                                                                                     `aagtgggAGGTAAATGGCTGGATGCCTAACTACGTGACAGCTTCGTTGCGAAGTCCAAGGCGTGGACCCAGGTTTGGGCTGGTAGGGCTTGCGGTGGGGTAAGTGTGACCGTTTCACCGGATCCTCGCTTCCGCAATATCCGGATTTAGTGTTCCAAAAACCCTTGGAACTTCTTCCGTTCCGGCCCCGATGGAATCTACTTCATAGCCCCTGATGGTGGACGGTTACGAATCCAAATACACTCCAACCGTCCCGGACTCAAGAATATTGCAGACCGTTGTTCAGTAACTTATGCcatcat                                                                                     >  1:138541/5‑301 (MQ=255)`  
                                                                                                                                                                                                                      `gTGGGGAGGTAAATGGCTGGATGCTTACCTACGTGACAGCTTCGTTGCGAAGTCCAAGGCGTGGACTCAGGTGTGGTCTGGTAGGGCTTGCGGTGGGGTAAGTGTGACTGTTTCACAGGATCTCCGCTTCCGCAATATCTGGATTAAGTGTGCCAACAACTCTTGGAACTTCTTCCGTACTGGCCCCGATGGAATCTACTTCATAGCCTCTGATGGTTGATGGTTACGAGTCCAAATACACTCCAACCGGCTCGGATTCAAGAAGATTGCAGAcctgtttcttatacacgactccgagccc                                                                                    >  1:467541/1‑274 (MQ=255)`  
                                                                                                                                                                                                                       `tGGGGAGGTAAATGGCTGGATGCTTACCTACGTGACAGCTTCGTTGCGAAGTCCAAGGAGTGGACTCAGGTGTGGTCTGGTAGGGCTGGCGGTGGGGTAAGTGTGACTGTTTCACAGGATCTCCGCTTCCGCAATATCTGGATTAAGTGTGCCAACAACTCTTGGAACTTCGTCCGTACTGGCCCCGATGGAATCTACTTCATAGCCTCTGATGGTGGATGGTTACGATTCCAAATACACTCCAACGGTCTCGGATTCAAGAATATTGCAGACAGTCGTTCAGTACCTAATGCAATCAtgg                                                                                   <  1:263737/301‑1 (MQ=255)`  
                                                                                                                                                                                                                          `ataagagacaggCTGGATGCTTACCTACGTTACAGTTTCGTTGCGAAGTCCAAGGCGTGGACTCAGGTGTGGTCTGGTAGGGCTGGCGGTGGGGTAAGTGTGACTGTTTCACAGGATCTCCGCTTCCGCAATATCTGGATTAAGTGTGCCAACAACTCTTGGAACTTCTTCCGTACTGGCCCCGATGGAATCTACTTCATAGCCTCTGATGGTGGATGGTTACGATTCCAAATACACTCCAACGGTCTCGGATTCAAGAATATTGCAGACAGTCGTTCAGTACCTAATGCAATCAtggtgg                                                                                <  1:272169/291‑1 (MQ=255)`  
                                                                                                                                                                                                                           `gAGGTAAATGGCTGGATGCTTACCTACGTGACAGCTTCGTTGCGAAGTCCAAGGCGTGGACTCAGGTGTGGTCTGGTAGGGCTGGCGGTGGGGTAAGTGTGACTGTTTCACAGGATCTCCGCTTCAGCAATATCTGGATTAAGTGTGCCAACAACTCTTGGAACTTCTTCCGTACTGGCCCCGATGGAATCTACTTCATAGCCTCTGATGGTGGATGGTTACGATTCCAAATACACTCCAACGGTCTCGGATTCAAGAATATTGCAGACAGTCGTTCAGTACCTAATGCAATAATGGTGga                                                                               >  1:434189/1‑301 (MQ=255)`  
                                                                                                                                                                                                                             `ggTAAATGGCTGGATGCTTACCTACGTGACAGCTTCGTTGCGCAGTCCAAGGCGTGGACTCAGGTGTGGTCTGGTAGGGCTGGCGGTGGGGTAAGTGTGACTGTTTCACAGGATCTCCGCTTCCGCAATATCTGGATTAAGTGTGCCAACAACTCTTGGAACTTCTTCCGTACTGGCCCCGATGGAATCTACTTCATAGCCTCTGATGGTGGATGGTTACGATTCCAAATACACTCCAACGGTCTCGGATTCAAGAATATTGCAGACAGTCGTTCAGTACATCATGctgtctcttatacac                                                                             >  1:10007/1‑287 (MQ=255)`  
                                                                                                                                                                                                                                     `gCTGGATGCTTACCTACGTGACAGCTTCGTTGCGAAGTCCAAGGCGTGGACTCAGGTGTGGTCTGGTAGGGCTGGCGGTGGGGTAAGTGTGACTGTTTCACAGGATCTCCGCTTCCGCAATATCTGGATTACGTGTGCCAACACCTCTTGGAACTTCTTCCTTACTGGCCCCGATGGAATCTACTTCATAGCCTCTGATGGTGGATGGTTACGATTCCAAATACACTCCACCGGTCTAGGATTCAAGAATATTGCAGACAGTCGTTCATTACCTAATGCAATCATGGTTGAGAACGAGTat                                                                     >  1:227650/1‑300 (MQ=255)`  
                                                                                                                                                                                                                                         `gATGCTTACCTACGTGACAGCTTCGTTGCGAAGTCCAAGGCGTGGACTCAGGTGTGGTCTGGTAGGGCTGGCGGTGGGGTAAGTGTGACTGTTTCACAGGATCTCCGCTTCCGCAATATCTGGATTAAGTGTTCCAACAACTCTTGGAACTTCTTCCGTACTGGCCCCGATGGAATCTACTTCATAGCCTCTGATGGTGGATGGTAACGATTCCAAATACACTCCAACGGTCTCGGATTCAAGAATATTGCAGACAGTAGTTCAGTACCTAATGCAATCATGGTGGAGAACGAGTAATTgg                                                                 <  1:208942/301‑1 (MQ=255)`  
                                                                                                                                                                                                                                          `aTGCTTACCTACGTGACAGCTTCGTTGCGAAGTCCAAGGCGTGGACTCAGGTGTGGTCTGGTAGGGCTGGCGGTGGGGTAAGTGTGACTGTTTCACAGGATCTCCGCTTCCGCAATATCTGGATTAAGTGTGCCAACAACTCTTGGAACTTCTTCCGTACTGGCCCCGATGGAATCTACTTCATAGCCTCTGATGGTGGATGGTTACGATTCCAAATACACTCCAACGGTCTCGGTTTCAAGCATATTGCAGACAGTCGTTCCGTACCTAATGCAATCATGGTGGAGTACGAGTAATTGGt                                                                >  1:39605/1‑301 (MQ=255)`  
                                                                                                                                                                                                                                             `cTTACCTACGTGACAGCTTCGTTGCGAAGTCCAAGGCGTGGACTCAGGTGTGGTCTGGTAGGGCTGGCGGTGGGGTAAGTGTGACTGTTTCACAGGATCTCCGCTTCCGCAATATCTGGATTAAGTGTGCCAACAACTCTTGGAACTTCTTCCGTACTGGCCCCGATGGAATCTACTTCATAGCCTCTGATGGTGTATGGTTACGATTCCAAATACACTCCAACGGTCTCGGATTCAAGAATATTGCAGACAGTCGTTCAGTACCTAATGCAATCactgtctcttatacacatctccgagc                                                             >  1:216592/1‑276 (MQ=255)`  
                                                                                                                                                                                                                                                     `cGTGACAGCTTCGTTGCGAAGTCCAAGGCGTGGACTCAGGTGTGGTCTGGTAGGGCTGGCGGTGGGGTAAGTGTGACTGTTTCACAGGATCTCCGCTTCCGCAATCTCTGGATTAAGTGTGCCAACAACTCTTGGAAATTCTTCCGTACTGGCCCCGATGGAATCTACTTCATAGCCTCAGAGGGTGGATGTTTACGAATACAAATACATTCAAGCGGCCTCGGATTCAAGAATATTGCAGACAGTCGTTCAGTACTGAATGCAATAAGGGTGGCGAACGAGAAATTGGTaaacaacatga                                                     >  1:313187/1‑293 (MQ=255)`  
                                                                                                                                                                                                                                                            `gCTTCTGTGCGAAGTCAAAGGAGTTGACTCAGGTGTGGTCTGGTAGGGCTTGCGGTGGGGTAAGTGTGACTGTTTCACAGGATCTCCGCTTCCGCAATATCTGGATTAAGTGTGCCAACAACTCTTGGAACTTCTTCCGTACTGGCCCCGATGGAATCTACTTCATAGCCTCTGATGGTGGATGGTTACGATTCCAAATACACTCCAACGGTCTCGGATTCAAGAATATTGCAGACAGTCGTTCAGTACCTAATGCAATCATGGTGGAGAACGAGTAATTGGTAAATCACAAGGAAAGACg                                              <  1:91419/301‑1 (MQ=255)`  
                                                                                                                                                                                                                                                                 `gTTGCGAGGGCCAAGGGGGGGAATCAGGGGGGGTATGGGAGGGCGTGCGGTGGGGTAAGTGTGACTGTTTCACAGGATCTCCGATTCCGGAATATCTGGATTAAGTGTGCCAACAACTCTTGGAACTTCTTCCGTACTGGCCCCGATGGAATCTACTTCATAGCCTCTGATGGTGGATGGTTACGATTCCAAATACACTCCAACGGTCTCGGATTCAAGAATATTGCAGACAGTCGTTCAGTACCTAATGCAATCATGGTGGAGAACGAGTAATTGGTAAATCACAAGGAAAGACGTGTAg                                         <  1:120137/301‑1 (MQ=255)`  
                                                                                                                                                                                                                                                                 `gTTGCGAAGTCCAAGGCGTGGACTCAGGTGTGGTCTGGTAGGGCTGGCGGTGGGGTAAGTGTGACTGTTTCACAGGATCTCCGCTTCCGCAATATCTGGATTAAGTGTGCCAACAACTCTTGGAACTTCTTCCGTACTGGCCCCGATGGAATCTACTTCATAGCCTCTGATGGTGGATGGTTACGATTCCAAATACACTCCAACGGTCTCGGATTCAAGAATATTGCAGACAGTCGTTCAGTACCTAATGCAATCATGGTGGAGAACGAGTAATTGGTAAATCACAAGGAAAGACGTGTAg                                         <  1:439034/301‑1 (MQ=255)`  
                                                                                                                                                                                                                                                                     `tgggtataagagacaggacgtaGGTGTGGTCTGGTAGGGCTGGCGGTGGGGTAAGTGTGACTGTTTCACAGGATCTCCGCTTCCGCAATATCTGGATTAAGTGTGCCAACAACTCTTGGAACTTCTTCCGTACTGGCCCCGATGGAATCTACTTCATAGCCTCTGATGGTGGATGGTTACGATTCCAAATACACTCCAACGGTCTCGGATTCAAGAATATTGCAGACAGTCGTTCAGTACCTAATGCAATCATGGTGGAGAACGAGTAATTGGTAAATCACAAGGAAAGACGTGTAGTCCa                                     <  1:162777/280‑1 (MQ=255)`  
                                                                                                                                                                                                                                                                         `gTCCAAGGCGTGGACTCAGGTGTGGTCTGGTAGGGCTGGCGGTGGGGTAAGTGTGACTGTTTCACAGGATCTCCGCTTCCGCAATATCTGGATTAAGTGTGCCAACAACTCTTGGAACTTCTTCCGTACTGGCCCCGATGGAATCTACTTCATAGCCTCTGATGGTGGATGGTTACGATTCCAAATACACTCCAACGGTCTCGGATTCACGAATATTGCAGACAGTCGTTCAGTACCTAATGCAATCATGGTGGAGAACGAGTAATTGGTAAATctgtctcttatacacatctccgagccc                                 >  1:129794/1‑275 (MQ=255)`  
                                                                                                                                                                                                                                                                         `gTCCAAGGCGTGGACTCAGGTGTGGTCTGGTAGGGCTGGCGGTGGGGTAAGTGTGACTGTTTCACAGGATCTCCGCTTCCGCAATATCTGGATTAAGTGTGCCAACAACTCTTGGAACTTCTTCCGTACTGGCCCCGATGGAATCTACTTCATAGCCTCTGATGGTGGATGGTTACGATTCCAAATACACTCCAACGGTCTCGGATTCAAGAATATTGCAGACAGTCGTTCAGTACCTAATGCAATCATGGTGGAGAACGAGTAATTGGTAAATCACAAGGAAAGACGTGTAgctgtctct                                 >  1:380088/1‑293 (MQ=255)`  
                                                                                                                                                                                                                                                                              `aGGCGTGGACTCAGGTGTGGTCTGGTAGGGCTGGCGGTGGGGTAAGTGTGACTGTTTCACAGGATCTCCGCTTCCGCAATATCTGGATTAAGTGTGCCAACAACTCTTGGAACTTCTTCCGTACTGGCCCCGATGGAATCTACTTCATAGCCTCTGATGGTGGATGGTTACGATTCCAAATACACTCCAACGGTCTCGGATTCAAGAATATTGCAGACAGTCGTTCAGTACCTAATGCAATCATGGTGGAGAACGAGTAATTGGTAAATCACAAGGAAAGACGTGTAgctgtctcttatac                            >  1:374381/1‑288 (MQ=255)`  
                                                                                                                                                                                                                                                                                    `ggACTCAGGTGTGGTCTGGTAGGGCTGGCGGTGGGGTAAGTGTGACTGTTTCACAGGATCTCCGCTTCCGCAATATCTGGATTAAGTGTGCCAACAACTCTTGGAACTTCTTCCGTACTGGCCCCGATGGAATCTACTTCATAGCCTCTGATGGTGGATGGTTACGATTCCAAATACACTCCAACGGTCTCGGATTCAAGAATATTGCAGACAGTCGTTCAGTACCTAATGCAATCATGGTGGAGAACGAGTAATTGGTAAATCACAAGGAAAGACGTGTAGTCCACGGactgtctcttat                      >  1:189070/1‑290 (MQ=255)`  
                                                                                                                                                                                                                                                                                           `ggTGTGGTCTGGTAGGGCTGGCGGTGGGGTAAGTGTGACTGTTTCACAGGATCTCCGCTTCCGCAATATCTGGCTTAAGTGTGCCAACAACTCTTGGAACTTCTTCCGTACTGGCCCCGATGGACTCTACTTCATAGCCTCTGATGTTGGATGGTTACGATTCCAAATACACTCCAACGGTCTCGGATTCAAGAATATTGCAGACAGTCGTTCAGTACCTAATGCAATCATGGTGGAGAACGAGTAATTTGTAAATCACAAGGAAAGACGTGTAGTCCACGGATGGACTCTCAAGGAGGTa               >  1:498964/1‑301 (MQ=255)`  
                                                                                                                                                                                                                                                                                           `ggTGTGGTCTGGTAGGGCTGGCGGTGGGGTAAGTGTGACTGTTTCACAGGATCTCCGCTTCCGCAATATCTGGATTAAGTGTGCCAAAAACTCTTGGAACTTCTTCCGTACTGGCCCCGATGGAATCTACTTCATAGCCTCGGATGGTGGATGGTTACGATTCCAAATACACTCCAACGGTCTCGGATTCAAGAATATTGCAGACAGTCGTTCAGTACCTAAGGCAATCATGGTGGAGAACGCGTACATGGGAAATCACAAGGAAAGAAGTGTAGTCCACGGAGGGACTCTCAAGGAGGTa               >  1:415573/1‑301 (MQ=255)`  
                                                                                                                                                                                                                                                                                             `tgtgGTCTGTTAGGGCGGGCGGTGGGGGAAGTGGGACTGTGTCACAGGATCTCCGCTTCCGAAATATCTGGATTAAGTGTGCCAAAAACTCTTGGAACTTTTGACGTACTGGCCGCGATGGAATCTACTTTATAGCCTCTGATGGTGGATGGTTACGATTCCAAATACACTCCAACGGTCTCGGATTCAAGAATATTGCAGACAGTCGTTCAGTACCTAAGGCAATCATGGTGGAGAACGAGTAATTGGTAAATCACAAGGAAAGACGTGTAGTCCACGGATGGACTCTCAAGGAGGTAcc             <  1:385524/301‑2 (MQ=255)`  

                                                                                                                                                                                                                                                                                                          `|`                                                                                                                                                                                                                                                                                                            
`CCGCATCCGACAAGGCGCACTATATTCTATCTAAAGATGGTAACAGGAATAACTGGTACATTGGTAGAGGGTCAGATAACAACAATGACTGTACCTTCCACTCCTATGTACATGGTACGACCTTAACACTCAAGCAGGACTATGCAGTAGTTAACAAACACTTCCACGTAGGTCAGGCCGTTGTGGCCACTGATGGTAATATTCAAGGTACTAAGTGGGGAGGTAAATGGCTGGATGCTTACCTACGTGACAGCTTCGTTGCGAAGTCCAAGGCGTGGACTCAGGTGTGGTCTGGTAGTGCTGGCGGTGGGGTAAGTGTGACTGTTTCACAGGATCTCCGCTTCCGCAATATCTGGATTAAGTGTGCCAACAACTCTTGGAACTTCTTCCGTACTGGCCCCGATGGAATCTACTTCATAGCCTCTGATGGTGGATGGTTACGATTCCAAATACACTCCAACGGTCTCGGATTCAAGAATATTGCAGACAGTCGTTCAGTACCTAATGCAATCATGGTGGAGAACGAGTAATTGGTAAATCACAAGGAAAGACGTGTAGTCCACGGATGGACTCTCAAGGAGGTACAAGGTGCTATCA  >  NC_001604.1/35756‑36352`

  

Alignment Legend

Aligned base mismatch/match (shaded by quality score): `ATCG/ATCG` `< 3 ≤` `ATCG/ATCG` `< 10 ≤` `ATCG/ATCG` `< 19 ≤` `ATCG/ATCG` `< 34 ≤` `ATCG/ATCG` `< 38 ≤` `ATCG/ATCG`

Unaligned base: `atcg`    Masked matching base: `atcg`    Alignment gap: `‑`    Deleted base: `‑`
